# Testing candidate genes linked to corolla shape variation of a pollinator shift in *Rhytidophyllum* (Gesneriaceae)

**DOI:** 10.1371/journal.pone.0267540

**Published:** 2022-07-19

**Authors:** Valérie Poulin, Delase Amesefe, Emmanuel Gonzalez, Hermine Alexandre, Simon Joly

**Affiliations:** 1 Département de Sciences Biologiques, Institut de Recherche en Biologie Végétale, Université de Montréal, Montréal, Canada; 2 Department of Human Genetics, Canadian Centre for Computational Genomics (C3G), McGill University, Montréal, QC, Canada; 3 Microbiome Research Platform, McGill Interdisciplinary Initiative in Infection and Immunity (MI4), Genome Centre, McGill University, Montréal, QC, Canada; 4 Montreal Botanical Garden, Montréal, Canada; University of Naples Federico II, ITALY

## Abstract

Floral adaptations to specific pollinators like corolla shape variation often result in reproductive isolation and thus speciation. But despite their ecological importance, the genetic bases of corolla shape transitions are still poorly understood, especially outside model species. Hence, our goal was to identify candidate genes potentially involved in corolla shape variation between two closely related species of the *Rhytidophyllum* genus (*Gesneriaceae* family) from the Antilles with contrasting pollination strategies. *Rhytidophyllum rupincola* has a tubular corolla and is strictly pollinated by hummingbirds, whereas *R*. *auriculatum* has more open flowers and is pollinated by hummingbirds, bats, and insects. We surveyed the literature and used a comparative transcriptome sequence analysis of synonymous and non-synonymous nucleotide substitutions to obtain a list of genes that could explain floral variation between *R*. *auriculatum* and *R*. *rupincola*. We then tested their association with corolla shape variation using QTL mapping in a F_2_ hybrid population. Out of 28 genes tested, three were found to be good candidates because of a strong association with corolla shape: *RADIALIS*, *GLOBOSA*, and *JAGGED*. Although the role of these genes in *Rhytidophyllum* corolla shape variation remains to be confirmed, these findings are a first step towards identifying the genes that have been under selection by pollinators and thus involved in reproductive isolation and speciation in this genus.

## Introduction

The rapid diversification of flowering plants is commonly thought to be associated with their adaptation to a wide range of pollinators for reproduction [1]. Indeed, for animal-pollinated species, floral adaptations to functional groups of pollinators often result in character specialization that could lead to reproductive isolation and speciation [2–4]. Many floral traits can be involved in these adaptations to pollinators, such as nectar composition, color, scent, and morphology [5] and many studies have shown that such traits are under strong selection pressure by pollinators [6, 7]. However, the specific genes on which selection acts are generally unknown [but see 8–10].

Recently, numerous studies have shed light on the genetic and molecular basis of floral traits, contributing to our understanding of how plants adapt and speciate [11–13]. However, the inception and subsequent variation of these traits are often controlled by a complex network of genes that has yet to be described for many groups [14, 15]. A useful approach to investigate the genetic bases of floral traits is to study the quantitative trait loci (QTL) responsible for a phenotype of interest as it can estimate the number of genomic regions involved in determining the trait of interest and quantify their effects on the phenotypic variance and identify cases of pleiotropy and epistasis [16, 17], and identify candidate genes of interest [18–20]. QTL mapping of floral traits has been performed in *Ipomopsis* [21], *Iris* [22], *Petunia* [23, 24], *Mimulus* [25–29], among others. Most studies on the evolution of floral traits stop after mapping the QTLs and they rarely investigate the genes underlying the QTLs [16]. Exceptions, however, contribute to a better understanding of the genetics of floral traits, such as the identification of an anthocyanin concentration gene in *Mimulus* [20] and genes involved in floral scent in *Petunia* [23] and *Nicotiana* [30, 31]. Although functional analyses and heterologous transformation experiments are required to confirm the role of a gene and selection experiments are needed to demonstrate its role in adaptation [32], association studies are important to narrow down on the genes underlying significant morphological variation and provide targets for future research.

Corolla shape is critical in ensuring reproductive success, but the genetics of corolla shape differentiation among species is still poorly understood. This structure is important for pollinator attraction and mechanical fit with the pollinator for pollen deposition but is also involved in the deterrence of unwanted visitors [1, 19]. To achieve these functions, corolla shape varies in size (length and width), number of petals and disposition, fusion, curvature and symmetry. The genetic basis of these corolla features was investigated in QTL studies [21, 22, 29, 33–35], which largely found that corolla shape is a complex trait with a polygenic architecture (i.e. several loci of small to moderate effects on the phenotype). However, very few studies identified genes responsible for corolla shape variation outside model species. One exception is a paper by Ding et al. [36] that discovered that a mutation in an actin gene was responsible for a mutant phenotype causing a reduction in the corolla tube width of *Mimulus lewisii*.

Although we know little of the genes involved in floral shape adaptation between species, the development of flowers has been well studied, if only in a few model organisms. The genetic processes implicated in petal morphogenesis mostly concerns its initiation and identity in the floral meristem [15]. For example, the well-established ‘ABC model’ describes a group of B-function transcription factors responsible for petal and stamen identity in *Arabidopsis thaliana* (APETALA 3 (AP3) and PISTILLATA (PI)) and *Antirrhinum majus* (DEFICIENS (DEF) and GLOBOSA (GLO)) [37, 38]. These flower organ identity genes were later found to have the same DNA-binding protein domain (MADS box) and to be able to dimerize and form complexes [39]. This led to the ‘Quartet Model’ that proposes a combinatory action for the organ identity genes [40, 41]. Substantial advances in flower development came from *A*. *majus* and led to the bilateral symmetry model [42–44]. In this molecular network, CYCLOIDEA (CYC) and DICHOTOMA (DICH) are TEOSINTE BRANCHED1-CYCLOIDEA-PROLIFERATING CELL FACTOR (TCP) genes that give dorsal identity to the petals and activate the RADIALIS (RAD) gene. RAD is a MYB transcription factor that negatively regulates DIVARICATA (DIV) to restrain its expression in the ventral domain [45]. All these models were accompanied and followed by numerous molecular studies, each one adding a piece to the regulation puzzle of petal morphogenesis. Furthermore, much progress has been made by looking at cellular division in the flower [46–48]. Indeed, the regulation in time and space of cellular division, elongation and differentiation is what gives rise to growth directionality, patterning and final corolla morphology [49].

While the recent genetic studies are important to acquire molecular data, refine genetic models, and understand the fine-scale genetic control of petal characteristics, they also give a restricted view of corolla shape regulation. One reason for this restricted view is that our information comes from a limited number of model species [50] and application of these models across the angiosperms is still a matter of discussion (‘shifting boundary’ [51] and the ‘fading borders’ [52, 53] ‘(A)BC model’ [54]). Another reason is that these developmental studies do not help to identify which genes have been particularly important for the diversification of species. Therefore, to link the genetics of floral shape variation with its impact on plant evolution, it seems essential to study floral variation in groups where we know it had an impact on speciation.

The *Gesneriaceae* family could be one such group as corolla shape and pollination strategies show extensive variation [55–58]. The subtribe *Gesneriinae* is of particular interest because it radiated into more than 80 species in the West Indies from a common ancestor [59, 60] approximatively 10 Ma [56]. These species show various pollination strategies (hummingbird, bats, moth, generalists) and there have been frequent transitions between strategies during the evolution of the group [61, 62] suggesting that pollination transitions have been an important driver of evolution for the *Gesneriinae*.

**Fig 1:**
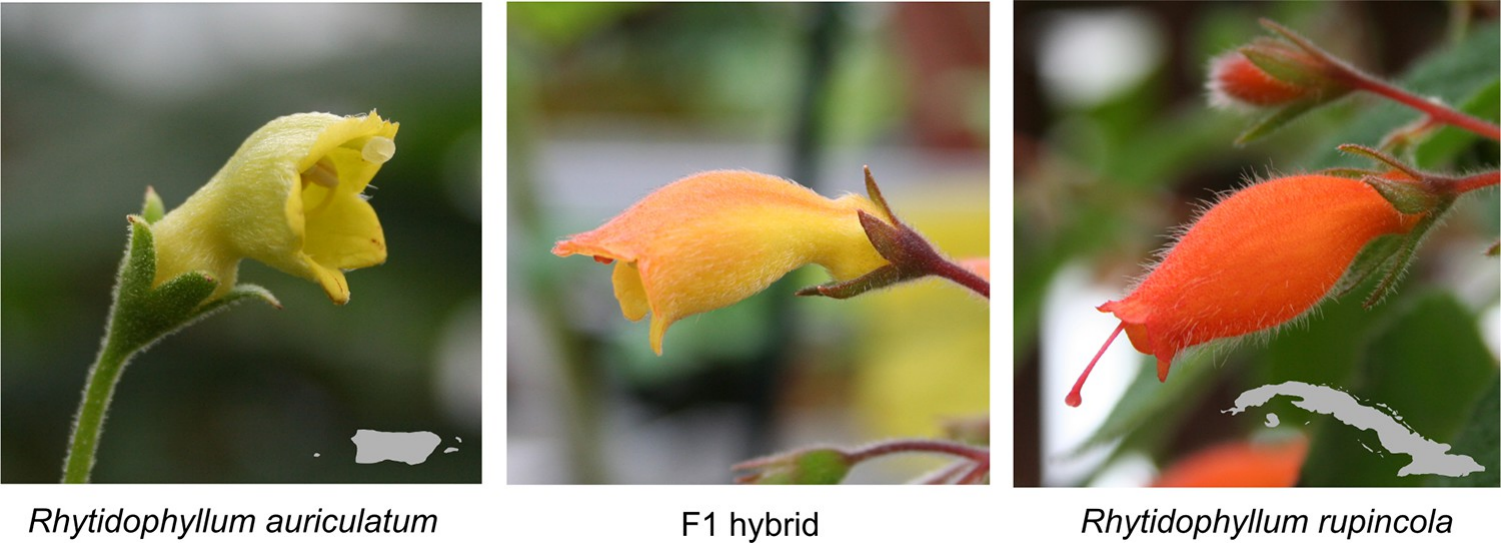
The two parental species studied. The small maps at the bottom-right corner of the images indicate their geographic origin. Photo credits: S. Joly.

Here, we investigate the genetic basis of the corolla shape differences between two representative species of the *Rhytidophyllum* genus from this subtribe: *Rhytidophyllum auriculatum* (Puerto Rico and Hispaniola) and *R*. *rupincola* (Cuba) (Fig 1). The first is a generalist, pollinated by hummingbirds, bats and insects, with yellow flowers that have a subcampanulate shape (bell shape with a basal constriction). The second, *R*. *rupincola*, is a hummingbird specialist with orange tubular flowers. We already know that the variation in corolla shape between *R*. *auriculatum* and *R*. *rupincola* is explained by a few QTLs of moderate to small effect [63]. However, we still have no idea which genes underly these QTLs and are thus responsible for the changes in flower shape between the two typical corolla morphotypes. Hence, the goal of this study was to identify candidate genes responsible for corolla shape variation between *R*. *auriculatum* and *R*. *rupincola*. This will help to better understand how flowers adapt to their pollinators, how transitions of pollination modes occur, and how floral traits evolve. We surveyed the literature and used a comparative transcriptome sequence analysis of synonymous and non-synonymous nucleotide substitutions to identify genes that could explain floral variation between *R*. *auriculatum* and *R*. *rupincola*. We then used transcriptome sequences to identify single nucleotide polymorphisms in these genes, genotyped them in a F_2_ hybrid population described in [63], and tested if these genes explain a significant amount of corolla shape variation between the species. Of the twenty-two genes investigated in this study, we identified 3 candidate genes that may underlie variation in corolla shape associated with pollination syndrome transition. We discuss these genes and their potential roles in determining corolla shape in *Rhytidophyllum*.

## Material and methods

### Transcriptome sequence comparison

Our objective was to compare the transcriptome sequences of *Rhytidophyllum rupincola* and *R*. *auriculatum* to identify genes associated with corolla shape determination that show evidence of directional selection. We assembled de novo the floral transcriptome of three *Rhytidophyllum* species with contrasting pollination strategies to identify candidate genes potentially involved with floral shape determination. In addition to *R*. *auriculatum* and *R*. *rupincola* described above, also included was *R*. *vernicosum*, an endemic species of the northern Dominican Republic that is bat pollinated with a corolla shape similar to *R*. *auriculatum*. *Rhytidophyllum auriculatum* and *R*. *vernicosum* are likely to form a monophyletic group [61, 62] and the addition of *R*. *vernicosum* in the transcriptome analysis increases the chance of identifying genes associated with the corolla shape because genes showing evidence of directional selection only with one of *R*. *auriculatum* or *R*. *vernicosum* could be discarded. All accessions used in this study come from the collections of the Montreal Botanical Garden: *Rhytidophyllum rupincola* (accession 113–1991), *R*. *auriculatum* (937–1971), and *R*. *vernicosum* (1267–1966).

Our initial objective was to obtain floral transcriptomes as complete as possible. Therefore, buds from all developmental stages as well as fully developed flowers were pooled prior to RNA extraction. Each sample thus contained multiple floral tissues such as sepals, petals, anthers, and the gynoecium. Buds were flash-frozen in liquid nitrogen immediately after harvest and were kept at -80 C until the RNA extraction.

Total RNA was extracted using a CTAB protocol [64] and RNA quantity and quality were assessed with a BioAnalyser (Agilent; Mississauga, Canada). mRNAs were extracted using poly-Toligo attached magnetic beads and cDNA synthesis was conducted using hexamer pairing. Illumina TrueSeq 100 bp paired-ends libraries were constructed (Illumina® TruSeq® RNA Sample Preparation Kit v1; San Diego, USA) and sequenced in a single lane of an Illumina HiSeq 2000 sequencing system at the Genome Quebec Innovation Centre (Montreal, Canada).

Raw reads were filtered for quality and for poor quality nucleotides at the beginning and end of each read using Trimmomatic vers. 0.32 [65] with the following parameters: “LEADING:15 TRAILING:15 SLIDINGWINDOW:5:15 MINLEN:40”. The Trinity software [66] was used to reconstruct de novo transcriptomes using default settings, discarding transcript < 201 bp. Sequences qualified as a “gene” were the union of transcripts similar enough to be considered by Trinity as putative isoforms of the same gene. These sequences were used for all further analyses. The quality of the transcriptomes was assessed with BUSCO v3 [67] against a set of single-copy plant orthologs (embryophyte_odb9).

Open reading frames (ORF) were obtained for each “gene” using Transdecoder [68], selecting the best ORF by transcript using results from a protein blast of the putative ORFs peptides on the uniref90 protein database [69]. Transcripts were annotated with Trinotate using protein and nucleotide blasts on the uniref90 protein database and Pfam annotation. Gene ontology (GO) annotations were extracted with Trinotate and functional enrichment tests were performed using the Bioconductor package GOseq [70].

Putative orthologous ORFs between species were obtained using OrthoMCL using default parameters [71]. Orthologous transcripts with a single sequence in all three species were aligned with MAFFT version 7.164 [72]. Synonymous and non-synonymous substitution rates were obtained between species for each ORF using the maximum likelihood method of Yang and Nielsen (2000) in PAML version 4.7 [73]. Alignments were trimmed to remove terminal stop codons.

### Identifying putative candidate genes involved in floral variation

We used two different approaches to a priori select genes that could be associated with corolla shape determination in *Rhytidophyllum*. First, among the orthologous genes with ORFs containing at least 100 synonymous sites (S), we extracted those with the greatest evidence of selection (dN/dS > 1) between the tubular-shaped corolla species *R*. *rupincola* and the two bell-shaped corolla species (*R*. *auriculatum* and *R*. *vernicosum*) and retained candidate genes associated with floral development GO-terms (S1 Table in S1 File).

For the second approach, we searched the literature and Gene Ontology [74] to find candidate genes potentially involved in floral shape variation. We thus searched for all the genes known to affect floral development in model species, or that were known to affect corolla or leaf shape. The main functions we were looking for were petal identity, bilateral symmetry, tissue polarity and petal growth regulation.

For each candidate gene, we obtained their nucleotide or amino acid sequences in databases such as Phytozome, UniProt or GenBank (see S2 Table for accession numbers in S1 File). We then searched for homologues in our transcriptomes using BLASTn in Geneious (version 8.1.9; Biomatters, Auckland, New Zealand) with an e-value cut-off of 1×10^−20^. Reciprocal BLASTn were done for each candidate transcript using GenBank to confirm the homology with their original candidate gene, which was deemed valid if the gene of origin was among the top 10 results (See S2 Table for details in S1 File). When multiple copies of the genes appeared to be present in the genomes of *Rhytidophyllum*, we included these in the survey. In such cases, a phylogenetic tree of the *Rhytidophyllum* gene copies and the original genes sequences was performed. Coding sequences were aligned using MAFFT vers. 7.450 [72] and a phylogenetic tree was reconstructed with RAxML vers. 8 [75] with a GTRGAMMAI nucleotide model (S1 Fig). For candidate genes to be considered further, transcripts for *R*. *auriculatum* (A) and *R*. *rupincola* (R) had to align with a minimum percent identity of 70%.

### SNP identification and validation

In order to genotype the candidate genes identified, we searched for single nucleotide polymorphisms (SNP) that differentiate the transcripts of the two parental species, *R*. *auriculatum* and *R*. *rupincola*. Transcripts were aligned in MAFFT (version 7.164 [72]) and putative SNPs were validated by PCR with primers located ca. 200 base pairs (bp) downstream and upstream of the SNP locations. For all the PCR reactions, the same master mix was used: 0.3 μl of DNA (ca. 1 to 10 ng of DNA) was added to 0.75 U of Dream Taq (Thermoscientific, Waltham, Massachusetts, USA), 1.5 μl of 10X Dream Taq Buffer, 0.6 μl of each 10 μM primers and 0.3 μl of 10 mM dNTPs in a total reaction volume of 15 μl. The amplification conditions were the same for all candidate genes, except for the annealing temperature (see S3 Table for details in S1 File): 2 min at 94°C, followed by 40 cycles of denaturation at 94°C for 15s, annealing at 48–54°C for 15s, elongation at 72°C for 30s, and a final extension step at 72°C for 1min. We PCR amplified 23 putative candidate genes from the two parental species and sequenced them using the Sanger platform at the Genome Quebec Innovation Centre (Montreal, Canada). The putative candidate gene sequences obtained were deposited in Genbank (see S4 Table for accession numbers in S1 File).

### Genotyping of the F_2_ hybrid population

The material genotyped consisted of extracted DNA from *Rhytidophyllum auriculatum*, *R*. *rupincola*, a first-generation (F_1_) hybrid from these parents and 173 individuals constituting a second-generation (F_2_) hybrid population obtained by selfing the F1 hybrid (see Alexandre et al. (2015) for more information). All candidate genes were genotyped using the Sequenom iPLEX Gold technology [76], which uses base-specific cleavage and mass spectrometry to genotype individuals in multiplex, by Genome Quebec. For six genes, two SNPs were genotyped resulting in 6 duplicates (differentiated by the suffix “.A” or “.B” after the gene name) (Table 1). Two putative candidate genes (NAC29.1 and HUA1) were also genotyped using PCR and enzymatic digestion as further validation. For this, DNA samples were amplified as described earlier (see S3 Table for primers in S1 File). For NAC29.1, 4 μL of the PCR amplicons were digested with NsiI (New England Biolabs, Whitby, Ontario, Canada), a restriction enzyme that overlap a SNP between parent species, in a total reaction volume of 15 μl according to the company’s protocol and digestion products were visualized on a 1% agarose gel. Genotyping of HUA1 was performed by migrating the PCR amplicons on agarose gel (1%) as the two parental alleles were of different lengths.

**Table 1:** Summary statistics of the three floral transcriptomes.

	R. auriculatum	R. rupincola	R. vernicosum
**Number of genes (Trinity clusters)**	66,955	67,396	70,662
**Number of transcripts**	164,127	166,900	165,516
**Median gene length (bp)**	923.5	910.2	899.7
**Mean gene length (bp)**	462	455	456
**Total nb. of ORFs**	95,149	96,916	91,424

### Linkage map construction

A linkage map was built with CarthaGene [77] using 557 Genotyping By Sequencing (GBS) markers from Alexandre et al. (2015), two putative candidate genes from the same study (CYCLOIDEA (CYC) and RADIALIS (RAD)), eight markers from the anthocyanin biosynthetic pathway (S. Joly, L. Fronteau, P.-A. Bourdon and H. Alexandre, unpublished data), and our 22 putative candidate genes (see results), for a total of 589 markers. Markers were divided into linkage groups with a maximum two points distance of 25 centimorgans (cM) between markers and a minimum LOD threshold of 3 using the *group* command. The *mrkdouble* and *mrkmerge* commands were used to eliminate possible double markers. After the groups were delimited, we used the *lkhd* function to order the markers in each linkage group using the 2-points distance optimization based on the Link-Kernighan heuristic algorithm. Then, for each linkage group, the best map was selected and the positions of markers in cM were obtained with the cumulative Haldane function.

### Phenotypic data

**Fig 2:**
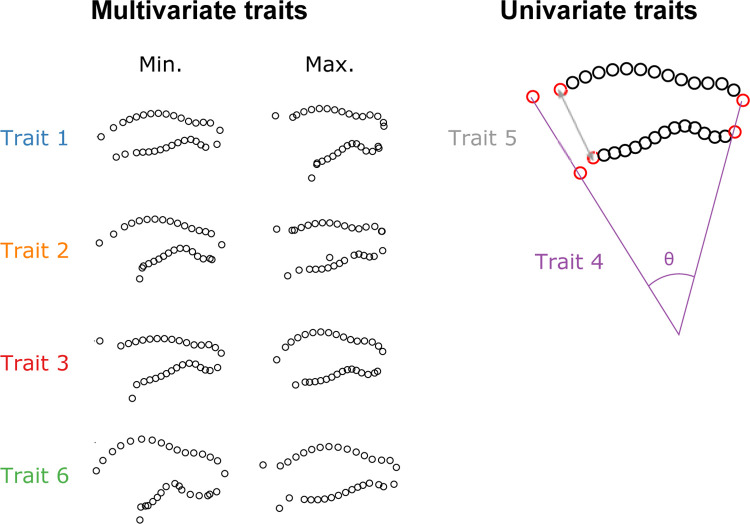
Schematic depiction of the phenotypic traits used in this study. For each multivariate trait, the whole range of corolla shape variation is represented by the landmarks (red dots) and semi-landmarks (black dots) of the corolla profile; the minimum and maximum shapes correspond to the most extreme values observed in the corresponding Principal Component Analysis (PCA) (see [63]). Under the univariate traits is an example of how measurements were performed from landmarks positions on a specific floral shape from landmarks (red dots).

**Fig 3:**
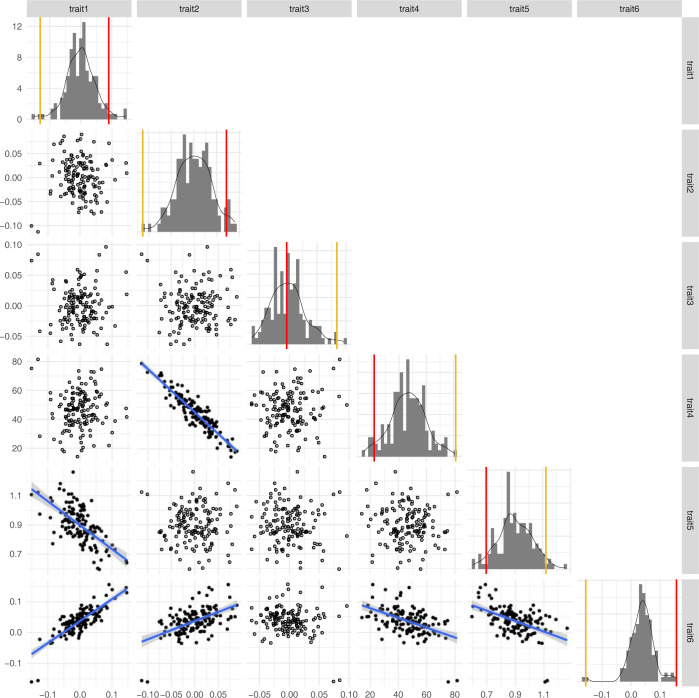
Morphological variation (diagonal) for the traits in the F_2_ hybrid population and pairwise scatterplots showing their correlation (lower diagonal). The yellow and red vertical lines on the histograms show the position of the parents, *R*. *auriculatum* and *R*. *rupincola*, respectively. Regression slopes are shown for traits comparisons with a significant Pearson correlation test (*p* < 0.05).

In order to obtain corolla shape quantitative trait loci (QTLs), we used the phenotypic data of Alexandre et al. [63] for six floral traits (Fig 2). These phenotypic data were available for 130 individuals among the 141 F_2_ hybrids that produced flowers. These six traits consist of a combination of geometric morphometric analyses and linear measurements, obtained using three approaches. First, the morphometric variation present among the F_2_ hybrids summarized by a principal component analysis (PCA) and the first three principal components (PC) that explain 71.9% of the variance (PC1: 35%, PC2: 22.7%, PC3: 14.2%,) were used for our QTL analysis (traits 1, 2 and 3 respectively). Broadly described, traits 1 and 3 explain variation in corolla opening (tube shape vs. campanulate shape) and petal lobe reflection, whereas trait 2 explains variation in corolla curvature (Fig 2). The second approach used landmarks data from the geometric morphometrics approach to obtain two univariate traits: corolla curvature (trait 4) and the corolla tube opening (trait 5). Finally, the third approach consisted of a PCA of the F_2_ individuals that included the two parents. We used the PC1 of this analysis that indicates the level of resemblance of hybrid individuals to each parent (Trait 6). Trait 6 summarizes information in corolla curvature, tube opening and petal reflection. There is redundancy in the shape variation between the different traits (Fig 3), but these alternative approaches increase the likelihood of detecting genes affecting the corolla shape variation.

### Quantitative trait loci detection

QTL mapping was performed to test an association between our putative candidate genes and corolla shape variation. QTL mapping was done in R Studio version 3.4.3 with the R/qtl package version 1.42–8 [78]. Phenotypic and genotypic data used for this study are accessible in the supplemental information. The recombination fractions between linkage groups were verified using *plotRF* function (see S2 Fig). We also searched for potential genotyping errors with the command *calc*.*errorlod* and marked the problematic genotypes (error LOD score >4) as ‘missing’ in the data before executing the QTL mapping. We finally looked for distorted segregation patterns of the markers using the function *geno*.*table*, that is genotypes with frequencies that departed significantly from the expected 1:2:1 ratio.

QTL mapping was done one trait at a time. Genotype probabilities were calculated every 1 cM with the function *calc*.*genoprob*. The probability of an association between the trait and the genotypes was calculated using the *scanone* function. The logarithm of odds (LOD) threshold above which an association was considered significant was determined with 10,000 permutations under the normal model and the Haley-Knott method. These thresholds values were calculated for alpha error thresholds of 5% and 10%. Only the sections of the linkage groups that scored higher than the LOD thresholds estimated by permutations were reported; these regions correspond to the position of the QTL. To obtain the confidence region of the QTL, the *lodint* command was used on the linkage group with the QTL using the default interval of 1.5 (below the LOD peak). To detect minor QTLs, we did the procedure again but with the primary QTL as a covariate in the model. After the all QTLs were detected for a phenotype, we used the *fitqtl* command to obtain the percentage of the variance explained (PVE) by the QTLs and their associated P-values. Interaction between QTLs was not included in the model and the variance was obtained by dropping one QTL at a time from the model. We tested for epistasis for each trait using the *scantwo* function of the rqtl package using Haley-Knott regression from multipoint genotype probabilities calculated at every 2.5 cM. A significance threshold of 0.05% was determined from 1000 simulations. We also calculated the LOD for all putative candidate genes to explain the morphological traits as well as probabilities of association using 10,000 permutations using the *scanone* function.

### Protein domain prediction and polymorphism localization

For genes that were associated with at least one shape QTL, we searched for SNPs or indels that could produce a protein-coding change between the sequences of *R*. *auriculatum* and *R*. *rupincola*. We looked for stop codons in the transcript sequences and for non-synonymous mutations in important protein domains. The domains were predicted from the transcriptome sequences by searching the CDD database using the Conserved Domains Search Service from NCBI [79]. Nucleotide insertions or deletion that caused a shift in the open reading frame of a gene for a species were validated by Sanger sequencing of gene amplicons obtained from genomic DNA or from floral bud RNA that was reversed-transcripted to DNA (SuperScript™ III Reverse Transcriptase, Thermo Fisher Scientific).

## Results

### Transcriptome sequence comparison

We obtained 60.1 × 10^6^, 58.5 × 10^6^, and 74.6 × 10^6^ raw reads for *R*. *auriculatum*, *R*. *rupincola*, and *R*. *vernicosum*, respectively. These were deposited in NCBI SRA under project PRJNA732582. The characteristics of the floral transcriptome of the three species are presented in Table 1. The BUSCO analyses on the Trinity gene transcripts suggest that the floral transcriptome of *R*. *rupincola*, *R*. *auriculatum* and *R*. *vernicosum* contained 80.3% (61.7%), 80.3% (60.2%) and 81.9% (61.7% complete) of a set of 1440 single-copy plant orthologous genes.

Selection for the best ORF for each “gene” resulted in 95,149 ORFs for *R*. *auriculatum*, 96,916 ORFs for *R*. *rupincola*, and 91,424 ORFs for *R*. *vernicosum*. The transcriptomes and their annotation are made available in a public repository [80].

**Fig 4:**
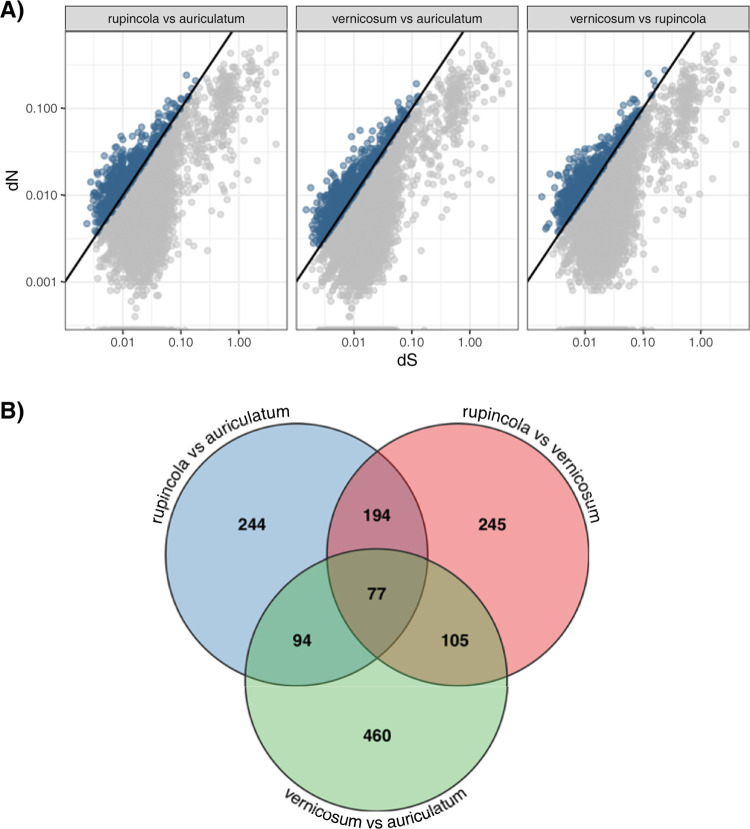
Comparative transcriptome sequence analysis of synonymous and non-synonymous nucleotide substitutions of the three *Rhytidophyllum* species. A) Plot of dN/dS ratio for each pairwise species comparisons. The line indicates the 1:1 slope and blue points represent open reading frames (ORFs) that have a dN/dS > 1. B) Venn diagram showing the ORFs that were found significant in the different pairwise comparisons in A.

The OrthoMCL analysis resulted in 16,066 clusters of putative homologous sequences between the two species. Of these, there were 9,435 clusters with one and only one homologous sequence in each genome. These were considered for the analyses of selection. After alignments and cleaning, 8,448 clusters could be properly analyzed for dN/dS ratios. A total of 1,419 genes had a dN/dS > 1 among all pairwise species comparisons (Fig 4). Of these, we focused on the 194 genes found to have a dN/dS >1 for both the *R*. *rupincola* / *R*. *auriculatum* and the *R*. *rupincola* / *R*. *vernicosum* comparison as these two comparisons involved tubular and bell shape corollas and thus were more likely to be involved in floral shape determination (Fig 4B). GO term enrichment analysis of these 194 genes compared to the genes represented in the homologous set suggested that the biological processes of “regulation of photoperiodism, flowering” (*p* = 0.0428) and “vegetative to reproductive phase transition of meristem” (*p* = 0.0192) were significantly over-represented.

### A list of twenty-eight genes potentially involved in floral variation

The comparison of transcriptome sequences identified seven putative candidate genes among the 194 selected above as having homology with a gene annotated to be involved in flower development. Most of these were transcription factors characterized in *Arabidopsis thaliana* or *Antirrhinum majus* known to be involved in floral development (S1 Table in S1 File).

The literature search resulted in a total of 28 genes, from which 21 were represented by pairs of homologous transcripts in *R*. *rupincola* and *R*. *auriculatum* (S2 Table in S1 File). Some genes had good homology with more than one pair of putative homologous transcripts, in which case we considered all pairs as different “putative candidate genes” and annotated them by numbers following the gene name (ex. GRXC7.1, and GRXC7.2). We thus obtained a list of 28 genes to test, 7 from the bioinformatic approach and 21 from the literature survey.

### Twenty-two genes were successfully genotyped

PCR amplification and Sanger sequencing were done on 23 of the 28 putative candidate genes. Of these, 20 genes had chromatograms of sufficient quality to validate the SNPs (S5 Table in S1 File). The remaining 8 genes were not validated but were still kept for genotyping; the sequences and SNP positions were based on the transcriptomes.

Thirty-seven markers were sent for genotyping by Sequenom iPLEX Gold, which included makers for 28 candidate genes, duplicate SNPs for six of these candidate genes (marked by suffix “.B”), RAD from Alexandre et al. (2015), U73C6, a putative anthocyanin pathway gene from an unpublished study, and NAC29.2, which was included by mistakenly in the genotyping. We obtained good genotype data for 30 of the 35 SNPs (S5 Table in S1 File). The markers that failed were CUC1, CUC2, CIN2A, DIV.2 and NAC29.2. Four more markers (two candidates and their duplicates) were eliminated because of problematic genotypic data: CYC1C.A, CYC1C.B, DIV.A and DIV.B. In the case of CYC1C.A and CYC1C.B, their SNPs were identified solely from the transcriptomic data and all F_2_ hybrids scored as homozygous for the *R*. *auriculatum* parent (AA). The two other markers DIV.A and DIV.B were discarded because *R*. *auriculatum* scored as homozygous for the *R*. *rupincola* parent allele (RR) and because the call reliability (or quality) was rated “fair” (rates of 88.57% and 88.00%). The SNPs used for these two markers were located in introns and as such were based only on Sanger sequences. Genotypic data for all other markers had call rates ranging from 97.7% to 100%, which is rated “excellent”, except for ER (candidate gene ERECT) that was rated “good” (see S1 File).

The gel genotyping for HUA1(PCR) and NAC29.1 (enzymatic digestion) confirmed the Sequenom results (100%) and these results were used for the linkage map instead of the results from Sequenom because they contained fewer missing data. For genes with two SNPs genotyped, the results were always identical and the SNP with fewer missing genotypes was kept for the linkage map construction: CYC.2.A, NAC54.B, PER53.A and PHAN.B (Table 1, S1 File).

In summary, we obtained good genotype data for 22 genes, bringing the total to 24 when including the genes RAD and CYC genotyped by Alexandre et al. (2015). An additional marker (U73C6) genotyped in the Sequenom approach was also used to build the linkage map. Five of the 24 genes had segregation patterns that deviated significantly from the expected 1:2:1 ratio (S6 Table in S1 File). We decided to keep these markers in our analyses as we believe the deviation was not caused by an error in genotyping and could add valuable information to the linkage map and QTL mapping.

### Linkage map and QTL mapping

The analysis of the 589 markers with CarthaGene resulted in 16 linkage groups that were concordant with the study of Alexandre et al. (2015). This is also very similar to the number of chromosomes of these species of n = 14. Our linkage map has a total length of 1772.5 cM with an average distance between markers of 3.75 cM (S7 Table in S1 File).

**Fig 5:**
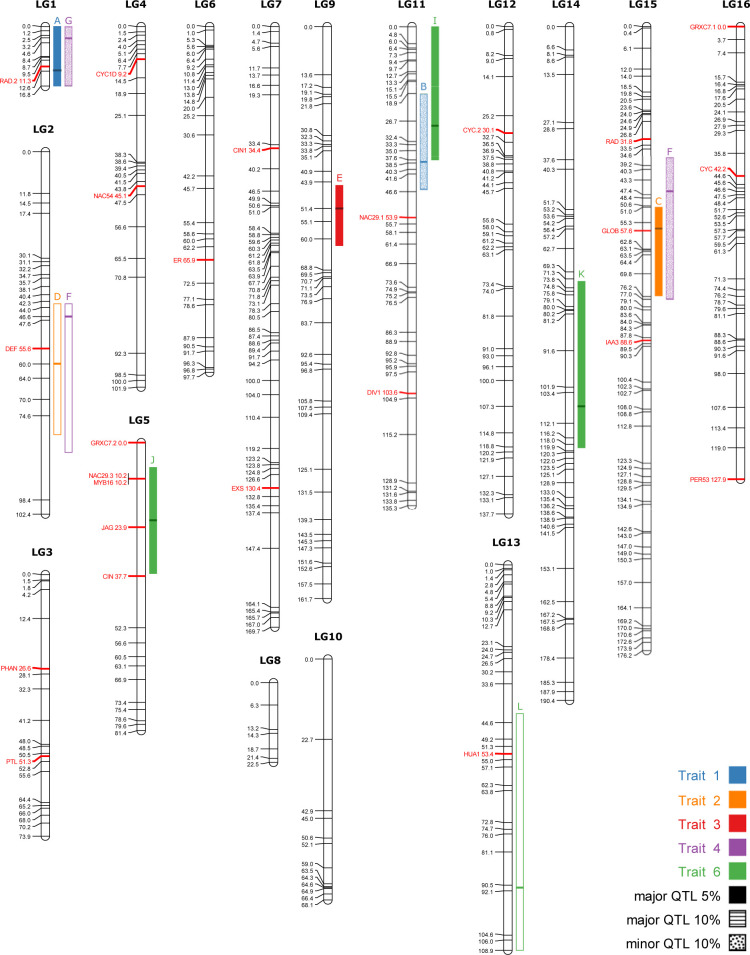
Linkage map with 12 QTLs. The linkage groups (LG) were given the same names as in Alexandre et al. [63] and QTLs are represented by coloured bars to the right of their designated linkage groups, with letters on top referring to Table 2. The patterns in QTLs bars represent different significance thresholds indicated in the legend, and the horizontal bar represents the peak location (see Table 2). The 24 candidate genes are in red, including CYC and RAD from Alexandre et al. [63]. Positions of markers are in cM. Minor QTLs are those that were detected after including the primary QTL as a covariate in the model.

QTL detection resulted in 9 primary and 3 minor QTLs, for a total of 12 (Table 2, Fig 5). Because some QTL from different traits colocalized, corolla shape was found to be associated with 8 distinct genomic regions on 8 linkage groups (Fig 5). All traits resulted in at least one QTL except for trait 5. In terms of percentage of variance explained (PVE), the individual QTLs ranged between 4.3% to 17% (mean: 10.5%) and the total variance explained per trait (excluding trait 5) varied between 15.2% to 43.0% (mean 27%) of variance explained (Table 2). No epistasis was detected for any trait (*p*<0.05).

**Table 2:** Information about the twelve QTLs identified.

Trait number and name	QTL	Linkage group	Peak position	Confidence region	Alpha	LOD threshold	Peak LOD score	Additive effect	Dominance effect	PVE	Total PVE	Alexandre et al. PVE
**1. Variation in the hybrids: PC1**	A	LG1	12.6	0.0–16.8	0.05	3.8	4.92	0.024 ± 0.005	0.008 ± 0.007	16.97%	26.24%	15.13%
	B	LG11	38	19–46	0.1	3.5	3.72	0.021 ± 0.005	0.001 ± 0.007	10.13%		
**2. Hybrids: PC2**	C	LG15	56	51–76	0.05	3.81	3.84	-0.007 ± 0.005	-0.022 ± 0.006	10.25%	22.81%	14.06%
	D	LG2	60	43–80	0.1	3.48	3.76	0.016 ± 0.004	-0.014 ± 0.006	10.00%		
**3. Hybrids: PC3**	E	LG9	51.4	45–62	0.05	3.88	4.63	-0.017 ± 0.004	0.001 ± 0.005	15.23%	15.23%	14.9%
**4. Corolla curvature**	F	LG2	46.6	43–85	0.1	3.48	3.77	-5.117 ± 1.443	6.244 ± 2.012	11.67%	28.64%	12.84%
	G	LG1	3.2	0.0–16.8	0.1	3.53	3.7	4.347 ± 1.484	2.215 ± 2.014	6.72%		
	H	LG15	56.0	37–77	0.1	3.53	3.58	1.915 ± 1.878	6.012 ± 2.139	5.99%		
**6. Parental differences**	I	LG11	28	0.0–37.6	0.05	3.85	3.86	0.016 ± 0.004	-0.001 ± 0.005	9.69%	43.02%	29.82%
	J	LG5	20	7–37,0	0.05	3.85	3.97	0.018 ± 0.007	0.003 ± 0.008	8.83%		
	K	LG14	107	72–119	0.05	3.85	4.03	0.015 ± 0.003	-0.012 ± 0.005	12.29%		
	L	LG13	91	42–108.9	0.1	3.49	3.76	0.011 ± 0.004	-0.003 ± 0.005	4.32%		

The letters attributed to each QTL for identification purposes are illustrated in Fig 5. Positions of confidence intervals (1.5 LOD score decrease) are given in centimorgan (cM) from the beginning of the linkage group. Alpha indicates the significance threshold at which the QTL was significant. PVE stands for the proportion of variance explained. Trait 5 did not result in any QTL.

We found that six candidate genes colocalized with at least one QTL (Fig 5). However, because the QTLs are large, we also estimated the probability that each of these genes is significantly associated with floral traits. Three genes had a logarithm of odds (LOD) greater than 3 (a 1000 to 1 ratio) to be associated with at least one corolla shape trait (Table 3). The only statistically significant result involves RAD.2 that is significantly associated with the PC1 of the hybrid population PCA (LOD = 4.58; *p* = 0.0093). Yet, JAG is marginally significantly associated with the PC1 of parental differences (LOD = 3.69; *p* = 0.061) and GLOBOSA is marginally significantly associated with the PC2 of the hybrid population PCA (LOD = 3.55; *p* = 0.087; Table 3).

**Table 3:** Candidate genes that have a logarithm of odds (LOD) greater than three (1000 to 1 odds) for an association with a floral trait, along with the statistical probability of association as determined by permutation of the linkage map.

Gene	Trait	LG	QTL type	Logarithm of odds (LOD)	p-value
RAD.2	1. Variation in the hybrids: PC1	1	Major	4.58	p = 0.0093
RAD.2	4. Corolla curvature	1	Minor	3.32	p = 0.16
JAG	6. Parental differences	5	Major	3.69	p = 0.061
GLO	2. Hybrids: PC2	15	Major	3.55	p = 0.087
GLO	4. Corolla curvature	15	Minor	3.43	p = 0.12

### Protein domain predictions

To identify candidate SNPs that could explain the variation in corolla shape in the seven genes that colocalized with the shape QTLs, we examined the ORFs for mutations that could affect the proteins they code for. No non-synonymous SNPs were detected in the conserved domains in any of the seven genes, nor any insertion or deletion that caused a shift in the open reading frames of the genes (S8 Table in S1 File).

## Discussion

The shape of corollas is critical for the efficient reproduction of many species. Yet, little is known of the genes involved in corolla shape determination apart from a few model species. Here we used a literature search and a transcriptome sequence comparison to identify putative candidate genes that might be involved in corolla shape transition between *R*. *rupincola* and *R*. *auriculatum*, two species that possess contrasting flower shapes. Although a QTL study previously outlined the genetic structure of the corolla for these species [63], nothing was known of the genes that may be involved in corolla shape modifications.

### The genetic structure of corolla shape transition between pollination strategies

The number of linkage groups found (16) and their size range (16.8 to 190.4 cM) are concordant with the linkage map obtained by Alexandre et al. [63] and we used the same linkage group numbers as in that previous study to facilitate comparisons. Despite the addition of 30 new markers (minus RAD and CYC which were included in the previous analysis), the two linkage maps were vastly concordant. The main difference relies in the longer length of LG5 due to the addition of the new markers, which contributed to a slightly longer total map length (1772.5 compared to 1650.6 cM). This extension of LG5 compared to Alexandre et al. is due to the addition of markers (GRXC7.2, NAC29.3, MYB16, JAG, CIN) that show significant segregation distortion (*p* << 0.01; S6 Table in S1 File), whereas markers with segregated distortion were automatically removed from the linkage map construction in Alexandre et al. It is not clear why all these markers located in this region of LG5 would be affected by segregation distortion.

Considering all phenotypic traits, eight genomic regions were found to be involved in floral shape determination; one more than obtained by Alexandre et al. [63]. This is caused by the loss of one QTL and a gain of two compared to this previous study. The LOD scores on LG16 where a QTL was lost for trait 5 are still relatively high, but not significant in this analysis (S3 Fig). The gain of two QTLs (C and J; Fig 5) appears to be due to the addition of new markers on the map. Still, many QTLs certainly remain to be detected as the 12 QTLs found in this study only explain a small to moderate portion of the variance of those traits (15.23% to 43.02% total variance explained for each trait). Nevertheless, the addition of new markers in this studied allowed to explain a greater proportion of the variance in the F_2_ population compared to the QTL study of Alexandre et al. (see Table 2).

Our results show that some shape QTLs co-localize onto a few linkage groups (LG1, LG11, LG15 and LG2; Fig 5, Table 2). On LG1, QTLs of traits 1 and 4 co-localize and cover the whole linkage group, which is the smallest. Two traits, 2 and 4, share more than one QTL as both have QTLs on LG15 and LG2. These two traits correspond to variation in corolla curvature (Fig 2) and are strongly correlated (Fig 3) highlighting that they essentially describe the same morphological variation. As such, it is reassuring that they identified the same genomic regions. However, QTLs for different traits also sometimes colocalized in the genome (e.g., traits 1 and 4 on LG1), which might suggest phenotypic integration, i.e. correlated variation of traits forming a functional unit [17, 81].

Our findings imply that there are several genes involved in flower shape variation with minor to moderate effect on the phenotype. This polygenic genetic architecture for floral shape was also found in *Mimulus* [82], *Leptosiphon* [33], *Iris* [22], *Ipomopsis* [21], *Penstemon* [34] and *Primulina* [35]. Though QTL studies on flower morphology often detect many loci dispersed in the genome, each explaining a small portion of the variance (reviewed in [16, 83]), this is not always the case. For instance, Bradshaw et al. [26] found at least one QTL of large effect (PVE >25%) for the majority of flower shape traits measured (7/9) while studying differences between sympatric species *Mimulus lewisii* and *M*. *cardinalis*. Another QTL study by Ferris et al. [29] on sympatric populations of *Mimulus guttatus* and *M*. *laciniatus* found 5 QTLs of large effect responsible for flower size and they suggested that ongoing geneflow could homogenize mutations of small effect. In our case, *Rhytidophyllum rupincola* (Cuba) and *R*. *auriculatum* (Hispaniola and Puerto Rico) live in allopatry and thus cannot exchange genes in the wild, which might have given more time for genes with small effects to contribute to the corolla shape differences between the two species.

Many things need to be considered when interpreting QTL studies, such as the number of markers and the size of the mapping population [16], but also whether the traits studied appropriately capture all the morphological variation [84]. A greater number of markers, in particular those that are likely to be associated with the trait of interest (the candidate genes), did seem to increase QTL detection in our study as well as the percentage of phenotypic variance explained. Yet, our small population size limits our capacity to detect QTLs of small effect and to precisely define the QTLs [63]. Indeed, some of our QTLs are quite large (up to 66.9cM), which increases the probability that colocalization between some candidate gene and QTLs could be only caused by chance.

### Molecular and biological functions of the three genes strongly associated with corolla shape variation

Although seven candidate genes co-localized with shape QTLs, only three of them stood as solid candidates by having a good probability of being associated with corolla trait variation (LOD > 3 and *p*-value < 0.1). These three genes were all derived from the literature search and have homology to well-known transcription factors from *A*. *majus* (GLO and RAD) or *A*. *thaliana* (JAGGED (JAG)). As these genes have been well studied in many angiosperm species, their functions in other organisms might provide information as to their role in the determination of floral shape differentiation in *Rhytidophyllum*.

GLO is a transcription factor from the MADS-box family that assume petal and stamen identity across angiosperms, albeit with few exceptions [85]. It can form heterodimers with DEFICIENS (DEF) and auto-regulate its expression. It has been shown that the relative expression levels of GLO and DEF differs during the developmental stages of *Antirrhinum* flowers and that this could explain the progression of the petal development, notably in the flower opening [86]. Expression patterns of GLO is asymmetric in *Maize* [87] and *Commelina* [88] flowers, suggesting a possible role in floral zygomorphy. Moreover, GLO gene duplications are associated with increasing perianth dimorphism in Zingiberales [89]. These evidences of association between GLO and corolla shape suggest that small differences in its expression level in *Rhytidophyllum* could influence corolla growth and shape inthis genus. Interestingly, the *Rhytidophyllum* putative GLO on LG15 was associated to QTLs for traits 2 and 4 that are linked with variation in corolla curvature. This could suggest that their effect on the phenotype is similar, which is concordant with their function described in the literature.

Another well studied gene in *A*. *majus* is RAD, a MYB transcription factor that plays a role in bilateral symmetry by promoting the dorsal identity of flowers [90]. The key factor that emerges from the zygomorphy genetic network is the asymmetrical expression pattern of the TCP and MYB transcription factors (bilateral symmetry genes) [91]. Many studies have investigated this genetic network in the order Lamiales [92–94], to which *Rhytidophyllum* belongs, and more specifically in the Gesneriaceae family [95–98]. Among the bilateral symmetry genes tested (6 including paralogs), only RAD.2 is associated with a QTL involved in floral morphology. It nevertheless represents a promising candidate gene because it is located near the peak of the QTL A on LG1 (Fig 5), explains the greatest proportion of variance in floral shape between the two parental species (Table 2) and is strongly associated with corolla trait 1 (Table 3) that describes overall corolla shape from bell-shaped corollas with large opening to tube-shaped corollas with narrow opening (Fig 2).

Finally, JAG is associated with trait 6 that combines variation in the position of the constriction at the base the corolla, curvature, as well as the reflection of the tip of the petals (Fig 2). JAG encodes a C_2_H_2_ type zinc finger protein that acts as a transcription factor (repressor and activator) to control lateral organ growth and patterning in *Arabidopsis* [99–102]. It is associated with cell proliferation in leaves, sepals and petals, with an overall effect on organ shape in *Arabidopsis* [99, 100], but also in *Oryza* [103], *Solanum* [104] and *Aquilegia* [105], suggestion a relatively preserved function across flowering plants. Its mechanism of action has been unravelled piece by piece over the years [106, 107]. Notably, Sauret-Güeto *et al*. [108] showed that JAG could increase the growth rate in the distal region of petals during their development, a function that could certainly impact corolla shape by controlling the growth and thus the width and length of the petals. In addition, JAG’s target genes show a broad range of action in many floral developmental process [102].

### Comparison of methods for the research of candidate genes

Of the two approaches used to find genes putatively involved in corolla shape differences in *Rhytidophyllum*, the literature search gave a longer list than the transcriptomics approach (21 vs 7). Our criterion for selecting genes under positive selection was very strict (dN/dS > 1), but lowering the threshold would have resulted in a large number of putative candidate genes. Other aspects limiting the power of this approach are the relatively few genes associated with floral development in the Gene Ontology database, that it could not consider genes that are not expressed at all in the flower tissues in one of the species, and that it only focusses on protein variants and as such will miss genes which expression is affected by cis-regulatory regions. Although this approach did not result in a strong candidate gene to explain corolla shape, it might represent an interesting approach to find sequences of interest in non-model species, especially when the genetic bases of a trait are not very well defined as with floral shape [109]. For future studies, differential gene expression, which was not possible here given the nature of our transcriptomes, might provide more candidates. In contrast, the literature approach resulted in 21 candidates that could be tested, of which 3 were found to have a strong association with corolla shape. Though certainly not exhaustive, the literature approach was successful in identifying very good candidates for genes involved in corolla shape determination in non-model species.

Candidate genes for determining inter-specific floral shape differences associated with distinct pollination strategies are rarely searched for. We show here that three genes known to be associated with corolla shape in other species are excellent candidates to be involved in floral shape differentiation and adaptation to different pollinator guilds in *Rhytidophyllum*. This study cannot confirm this relationship and these genes may also only be under linkage disequilibrium with a gene involved in corolla shape differentiation. Further validation through finer mapping or transformation is required.

In conclusion, we found that the literature search and the comparison of transcriptome sequences approaches, followed by QTL mapping on a F_2_ population, represents an interesting approach to obtain a list of candidate genes putatively involved in corolla shape associated with a pollinator shift in non-model species. This approach can later be integrated with other methods to fully understand the evolution of flower morphology in *Rhytidophyllum* and helps to understand the great floral diversification in this genus.

## Supporting information

S1 Fig Phylogenies of the copies of the genes CIN (A), CUC (B), CYC (C), and DIV (D) found in *Rhytidophyllum* and included in the study along with the reference sequences. The species name, the gene name and the GenBank accession number are indicated for each sequence.
(PDF)

S2 Fig Recombination fractions between linkage groups. (PDF)

S3 Fig Graphs of the LOD scores for the traits along the chromosomes. (PDF)

S1 File Supplementary information tables 1 to 8. (XLSX)
