# Supplementary figures and images for: Testing candidate genes linked to corolla shape variation of a pollinator shift in Rhytidophyllum (Gesneriaceae)
Source: PLoS One. 2022 Jul 19;17(7):e0267540. doi: 10.1371/journal.pone.0267540 (PMC9295946; doi:10.1371/journal.pone.0267540)

**A) CIN**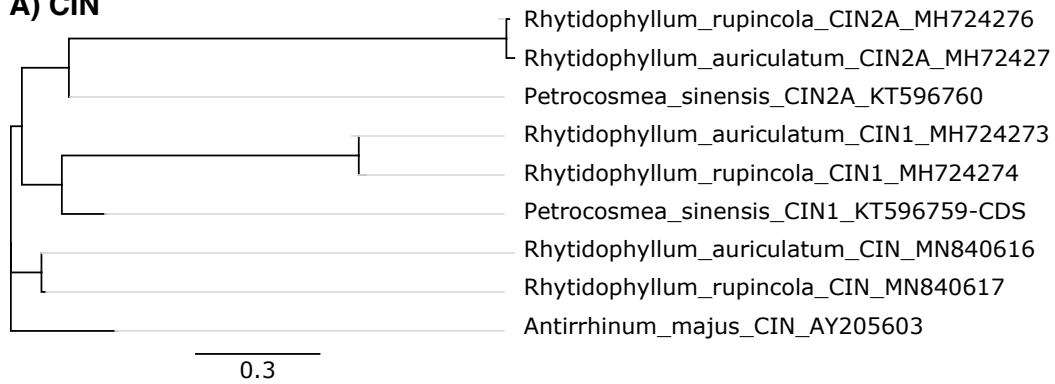**B) CUC**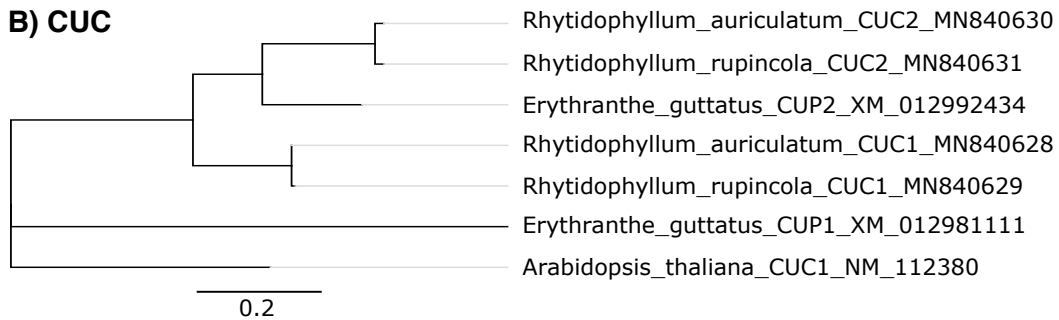**C) CYC**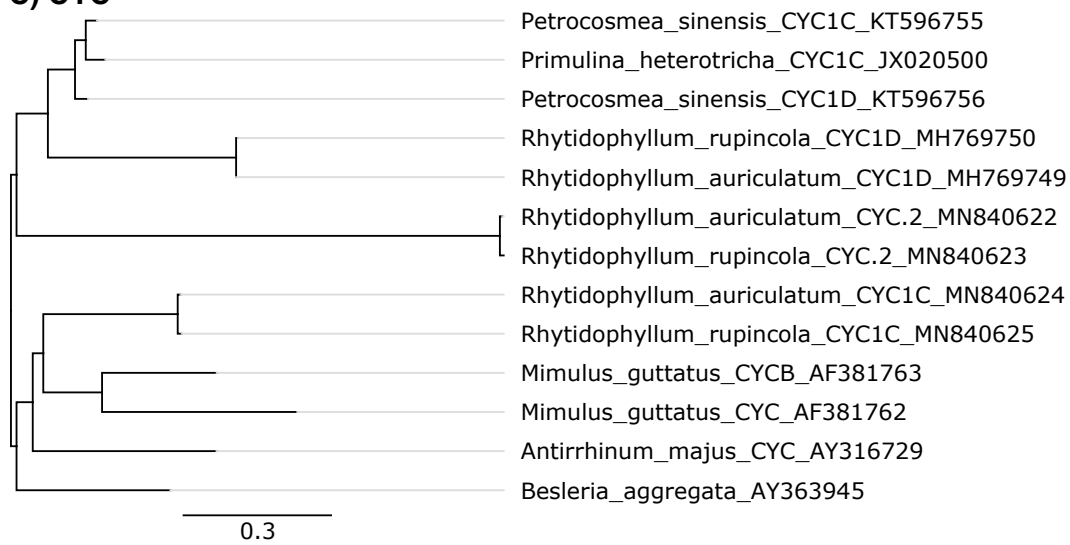**D) DIV**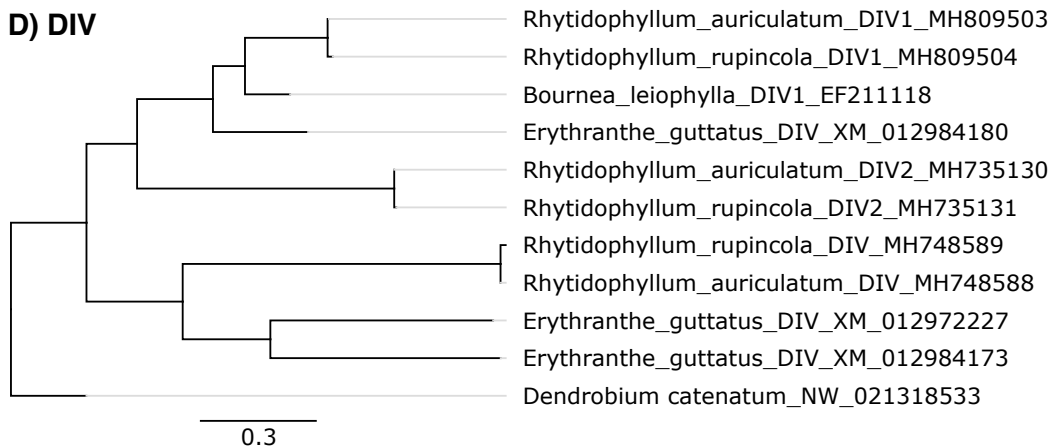

Supplement: S1 Fig — Phylogenies of the copies of the genes CIN (A), CUC (B), CYC (C), and DIV (D) found in Rhytidophyllum and included in the study along with the reference sequences. The species name, the gene name and the GenBank accession number are indicated for each sequence. (PDF) [file pone.0267540.s001.pdf]

Pairwise recombination fractions and LOD scores

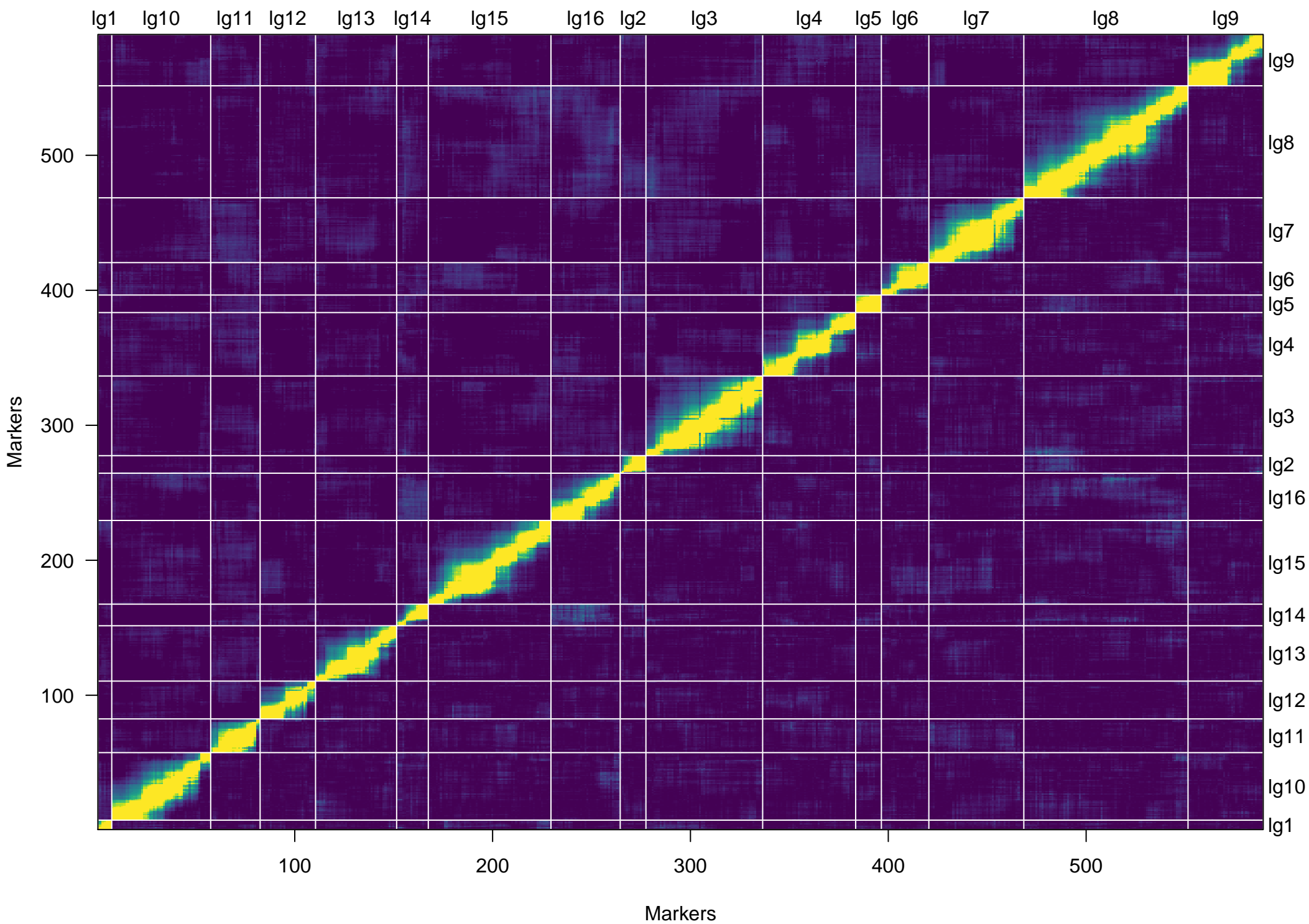

Supplement: S2 Fig — (PDF) [file pone.0267540.s002.pdf]
