# Supplementary material for: Testing candidate genes linked to corolla shape variation of a pollinator shift in Rhytidophyllum (Gesneriaceae)
Source: PLoS One. 2022 Jul 19;17(7):e0267540. doi: 10.1371/journal.pone.0267540 (PMC9295946; doi:10.1371/journal.pone.0267540)
Supplement: S3 Fig — (PDF) [file pone.0267540.s003.pdf]

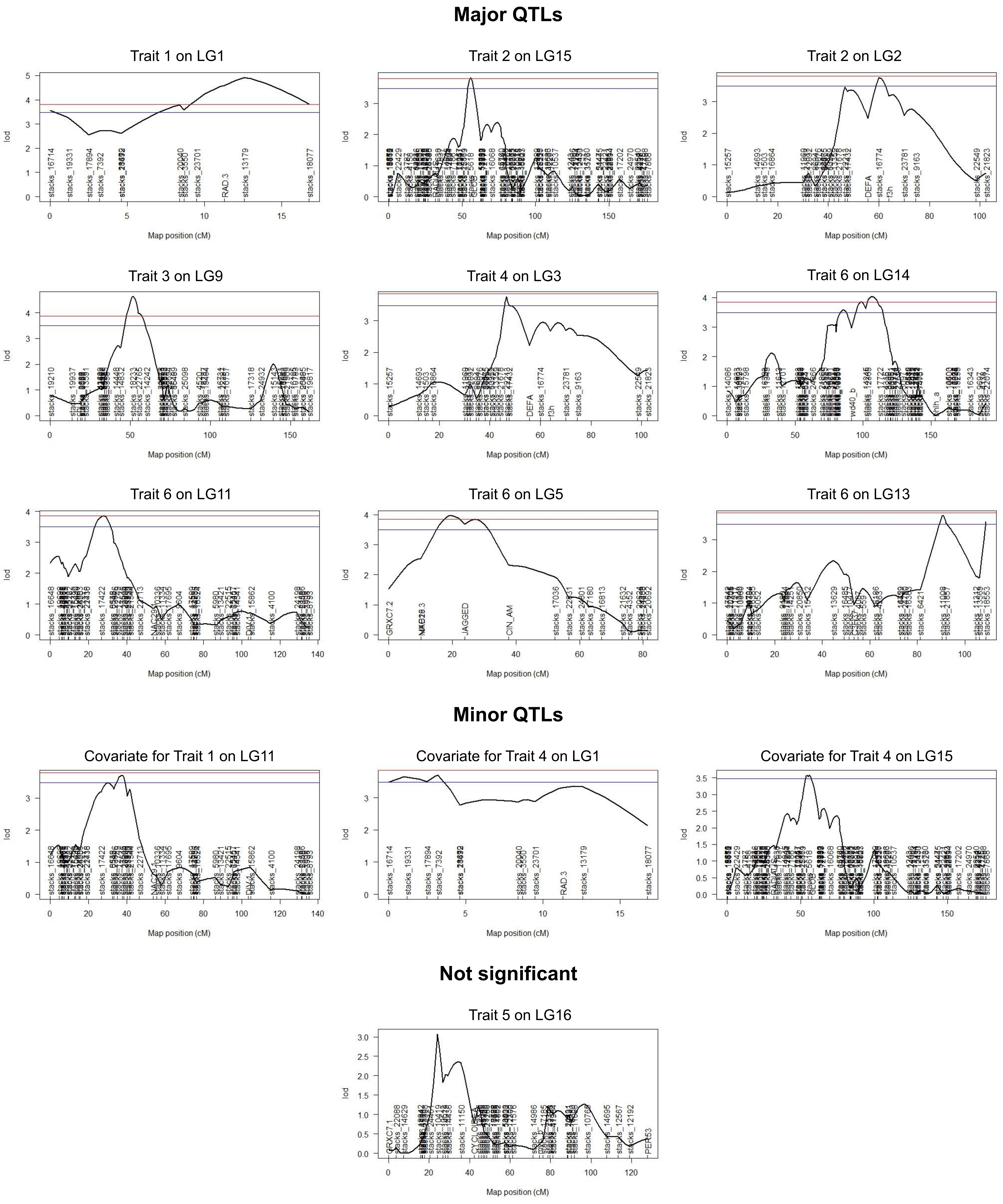
Major QTLs
Trait 1 on LG1
Trait 2 on LG15
Trait 2 on LG2
Trait 3 on LG9
Trait 4 on LG3
Trait 6 on LG14
Trait 6 on LG11
Trait 6 on LG5
Trait 6 on LG13
Minor QTLs
Covariate for Trait 1 on LG11
Covariate for Trait 4 on LG1
Covariate for Trait 4 on LG15
Not significant
Trait 5 on LG16
lod
Map position (cM)
